# From bench to bedside: elucidating VEGF(R) inhibitor-related heart failure in cancer treatment

**DOI:** 10.1186/s12967-025-06133-x

**Published:** 2025-01-23

**Authors:** Shengkun Peng, MinHong Cai, Hongyu Kuang, Anqi Lin, Qinghua Ma, Xiaoqin Dai, Peng Luo, Yijun Liu, Guo Zhang, Yifeng Bai

**Affiliations:** 1 Department of Radiology, Sichuan Provincial People’s Hospital, University of Electronic Science and Technology of China, Chengdu, 610072 Sichuan China; 2 Healthcare-Associated Infection Management Office, Sichuan Academy of Medical Sciences and Sichuan People’s Hospital, Chengdu, Sichuan China; 3 Department of Cardiology, University-town Hospital of Chongqing Medical University, Chongqing, China; 4 Department of Cardiology, The Second Affiliated Hospital of Chongqing Medical University, Chongqing, China; 5 Department of Oncology, Zhujiang Hospital, Southern Medical University, Guangzhou, China; 6 Department of Pediatrics, Sichuan Provincial People’s Hospital, School of Medicine, University of Electronic Science and Technology of China, Chengdu, China; 7 Department of Traditional Chinese Medicine, Sichuan Provincial People’s Hospital, School of Medicine, University of Electronic Science and Technology of China, Chengdu, China; 8 Department of Outpatient, Sichuan Provincial People’s Hospital, University of Electronic Science and Technology of China, Chengdu, China; 9 Department of Dean’s office, Sichuan Provincial People’s Hospital, School of Medicine, University of Electronic Science and Technology of China, Chengdu, China; 10 Department of Oncology, Sichuan Provincial People’s Hospital, University of Electronic Science and Technology of China, Chengdu, China

**Keywords:** Vascular endothelial growth factor, Vascular endothelial growth factor receptor, VigiBase, VEGF(R) inhibitor-related heart failure, Mouse model

## Abstract

**Background:**

Vascular endothelial growth factor (VEGF) and VEGF receptor (VEGFR) inhibitors play a pivotal role in treating various tumors; however, the clinical characteristics and molecular mechanisms of their associated heart failure (HF) remain incompletely understood.

**Methods:**

We investigated the epidemiological characteristics of VEGF or VEGFR inhibitors [VEGF(R)i]-related heart failure (VirHF) using the global pharmacovigilance database Vigibase. The phenotypic features and molecular mechanisms of VirHF were characterized using VEGF(R)i-treated mouse models through a combination of echocardiography, histopathological analysis, and transcriptome sequencing. Furthermore, we performed a retrospective analysis of cardiac function parameters in patients undergoing VEGF(R)i treatment at local hospitals.

**Results:**

In the analysis of 1871 VirHF cases, elderly patients (≥ 65 years) and female subjects demonstrated an elevated risk of occurrence. Experimental studies in mice revealed that both acute and chronic VEGF(R)i administration resulted in reduced left ventricular EF, cardiomyocyte hypertrophy, and myocardial fibrosis. Transcriptomic analysis identified significant dysregulation of multiple key signaling pathways, including DNA repair (R = 0.46), mitochondrial ATP synthesis (R = 0.39), glycogen metabolism regulation (R = 0.45), and proteasome-mediated protein degradation (R = 0.45). Moreover, significant upregulation was observed in inflammatory pathways, specifically those involving IL-1, IL-6, TNF-α, and IRF3/IRF7-mediated immune responses. Clinical cohort analyses demonstrated significant elevations in both cardiac injury biomarkers (NT-proBNP, CK-MB, cTnT) and inflammatory mediators (CRP) following VEGF(R)i administration.

**Conclusions:**

Our findings present the first comprehensive characterization of VirHF clinical features and elucidate its underlying molecular mechanisms, thereby providing a theoretical framework for optimizing the clinical safety of VEGF(R)i therapy.

## Introduction

The vascular endothelial growth factor (VEGF) or VEGF receptor (VEGFR) serve as a crucial regulator of tumor angiogenesis, and the development of its inhibitors [VEGF(R)i] has fundamentally transformed therapeutic strategies for diverse tumors [1]. Following the approval of bevacizumab for metastatic colorectal cancer treatment in 2004, numerous VEGF(R)i, including sorafenib and sunitinib, have been approved for the clinical treatment of multiple malignancies, particularly renal cell carcinoma and hepatocellular carcinoma [2]. Extensive clinical research data have demonstrated that VEGF(R)i significantly enhance patients' progression-free survival and overall survival, thus establishing novel treatment options for patients with advanced cancer [3]. Among breast cancer patients, bevacizumab treatment demonstrated significant improvement in median survival [hazard ratio (HR) = 0.71] [4]. In patients with advanced hepatocellular carcinoma, sorafenib demonstrated a significant increase in median survival (HR = 0.69) [5]. Additionally, in patients with pancreatic neuroendocrine tumors, sunitinib demonstrated significant enhancement of progression-free survival (HR = 0.42) [6]. Clinical trials in non-small cell lung cancer demonstrated that bevacizumab significantly extended overall survival time (HR = 0.79) [7].

The widespread clinical application of VEGF(R)i has garnered increasing attention from clinicians regarding their associated adverse events. Extensive research has demonstrated that VEGF inhibitors induce various adverse events, including but not limited to hypertension, proteinuria, hemorrhage and thrombosis, fistula formation, intestinal perforation, and posterior reversible encephalopathy syndrome (PRES) [2, 8]. Moreover, the off-target effects of VEGFR inhibitors (VEGFRi) [2] can trigger a broader spectrum of adverse drug reactions, such as fatigue, hypothyroidism, hand-foot syndrome, diarrhea, and hair changes [2, 9]. A recent pharmacovigilance study has not only revealed a significant increase in the incidence of VEGF(R)i-associated hypertension, but also elucidated its potential biological mechanisms [10], thereby providing crucial evidence for the clinical management of VEGF(R)i-related hypertension. Therefore, a comprehensive understanding of VEGF(R)i-induced adverse events and their biological mechanisms remains crucial for improving cancer patient prognosis and clinical outcomes.

Heart failure (HF) represents a severe and potentially life-threatening cardiovascular adverse event that significantly impacts the prognosis and quality of life of cancer patients. Large-scale retrospective cohort studies have demonstrated that cancer patients with concurrent HF exhibit a significantly increased mortality risk within 10 years following initial diagnosis [11]. With the expanding clinical implementation of novel antineoplastic agents, the incidence of drug-related HF has demonstrated an increasing trend. Extensive research has confirmed that various antineoplastic agents, specifically anthracyclines, immune checkpoint inhibitors, and VEGF(R)i, are associated with an increased risk of HF [12]. Although VEGF(R)i have significantly improved the prognosis of patients with various solid tumors, clinical evidence indicates that they substantially increase the risk of HF in patients [13]. Currently, systematic research on the clinical characteristics and molecular mechanisms of VEGF(R)i-related heart failure (VirHF) remains limited. Furthermore, real-world studies investigating the incidence, risk factors, prognostic implications, and biological mechanisms of VirHF remain notably scarce.

Building upon the previously described research background, this study implemented a comprehensive, multidimensional research strategy to systematically investigate the characteristics and potential mechanisms of VirHF. First, we systematically analyzed the epidemiological characteristics, risk factors, and prognostic manifestations of VirHF using data from the global spontaneous adverse reaction reporting database VigiBase. Second, we developed VEGF(R)i-treated mouse models and, in conjunction with transcriptome sequencing technology, comprehensively explored the molecular mechanisms of VirHF. Finally, through analysis of clinical data from local hospitals, we evaluated the impact of VEGF(R)i on cardiac function in cancer patients. The findings of this study not only advance our understanding of the mechanisms underlying VirHF but also provide crucial theoretical foundations for risk prevention and individualized medication strategies in clinical practice.

## Methods

### Adverse events data sources and processing

This pharmacovigilance study was conducted through comprehensive analysis of VigiBase, a global adverse drug reaction database. VigiBase consolidates spontaneously reported adverse drug reactions from more than 130 participating countries globally [14]. All adverse reaction records associated with VEGF(R)i therapy were systematically extracted and analyzed from the database spanning from January 1968 through December 2023. The association between VEGF(R)i and HF adverse reactions was evaluated using the Medical Dictionary for Regulatory Activities (MedDRA) standardized terminology [15], with the strength of association quantified through reporting odds ratio (ROR) and corresponding 95% confidence intervals [16]. Statistical significance for the association between specific HF adverse events and VEGF(R)i use was established based on predefined criteria: a minimum of three reported cases and a lower bound of the 95% confidence interval for ROR (ROR025) exceeding 1.

### Assessment of cardiac function parameters in cancer patients before and after VEGF(R)i therapy

We performed a retrospective analysis of solid tumor patients receiving VEGF(R)i therapy at Zhujiang Hospital of Southern Medical University, with systematic collection of biomarker data indicative of myocardial injury, including cardiac-specific markers such as creatine phosphokinase-MB (CK-MB), creatine phosphokinase (CK), N-terminal B-type Natriuretic Peptide (NT-proBNP), Cardiac Troponin T (cTnT), and the inflammatory biomarker C-Reactive Protein (CRP). The effects of VEGF(R)i therapy on cardiac function were evaluated by comparing mean changes in these parameters before and after treatment initiation in individuals. This study protocol was approved by the Institutional Ethics Committee of Zhujiang Hospital of Southern Medical University.

### Development and implementation of a VirHF mouse model

For this study, forty-eight male C57BL/6J mice (aged 6–8 weeks, with a mean body weight of 25 ± 0.5 g) were utilized. The mice were housed in a controlled environment (temperature and humidity regulated) with unrestricted access to standard laboratory chow and purified water. The study protocol was approved by the Institutional Animal Care and Use Committee of the Second Affiliated Hospital of Chongqing Medical University (ID: IACUC-SAHCQMU-2024-00174). Two targeted therapeutic agents were investigated: bevacizumab, a VEGF inhibitor (catalog no. 216974-75-3), and semaxanib, a VEGFR inhibitor (catalog no. 204005-46-9). Both compounds were obtained from MCE Pharmaceutical Technology Company. Phosphate-buffered saline (PBS) served as the vehicle for bevacizumab administration, while dimethyl sulfoxide (DMSO) was utilized for semaxanib preparation. The experimental animals were randomly allocated into two main groups: chronic cardiac toxicity (CCT, n = 24) and acute cardiac toxicity (ACT, n = 24). The CCT group was further subdivided into four groups: PBS control (n = 6), bevacizumab intervention (n = 6), DMSO control (n = 6), and semaxanib intervention (n = 6). In the CCT group, bevacizumab (5 mg/kg) [17] and semaxanib (10 mg/kg) [18] were administered twice weekly in 200 μl volumes over a 4-week period. The ACT group was similarly subdivided into four parallel groups, with doubled dosages (bevacizumab 10 mg/kg, semaxanib 20 mg/kg), maintaining identical administration frequency and volume over a 2-week intervention period.

### Cardiac function assessment, histopathological evaluation, and transcriptomic analysis in murine models

Experimental animals were anesthetized with 2.5% isoflurane and subjected to noninvasive cardiac function assessment using a VINNO6LAB color Doppler ultrasound imager with M-mode echocardiographic image acquisition. Cardiac function parameters, including left ventricular ejection fraction (EF) and fractional shortening (FS), were quantified from the echocardiographic measurements. Upon study completion, animals were euthanized, and heart and lung tissues were carefully excised and weighed to determine heart weight (HW) and lung weight (LW). Organ-to-body weight ratios (HW/BW, LW/BW) were subsequently calculated using the terminal body weight (BW). Sub-apical cardiac tissue specimens were fixed in 4% paraformaldehyde solution at room temperature (23 ± 2 °C) for 24 h, followed by sequential ethanol dehydration and paraffin embedding. Paraffin-embedded myocardial tissues were serially sectioned at 5-μm thickness and processed for histological analyses, including hematoxylin–eosin (H&E) staining, wheat germ agglutinin (WGA) immunofluorescence, Masson's trichrome staining, and Sirius Red staining. Histological images were acquired using a high-resolution microscopy system. Additional myocardial tissue specimens were processed for transcriptomic analysis using RNA sequencing.

### Molecular pathogenesis of VirHF

To investigate the underlying molecular mechanisms of VirHF, we performed a comprehensive pan-cancer transcriptomic analysis utilizing data from The Cancer Genome Atlas (TCGA) database. Single-sample gene set enrichment analysis (ssGSEA) was conducted using the Gene Set Variation Analysis (GSVA) software package [19]. Utilizing pathway annotations from Gene Ontology (GO), Kyoto Encyclopedia of Genes and Genomes (KEGG), and Reactome databases within MSigDB [20–23], we systematically assessed the activation states of diverse signaling pathways. By analyzing the associations between VirHF susceptibility and pathway activation patterns across multiple cancer types, we established a comprehensive molecular mechanism network.

### Statistical analysis

Statistical analyses were conducted using R (version 4.1.0) and GraphPad Prism (version 9.0) software packages. For skewed data distributions, non-parametric analyses (Mann–Whitney U test) were implemented; categorical variables were evaluated using chi-square tests or Fisher's exact tests when appropriate. The temporal characteristics of VirHF were analyzed and characterized using cumulative distribution curves. Univariate and multivariate logistic regression models were utilized to assess risk factors associated with patient prognosis. Experimental data from murine models were derived from six independent samples, and results were expressed as mean ± standard error of the mean (SEM). Sample sizes (n) for each experimental group were explicitly documented, with n denoting the number of biological replicates as opposed to technical replicates. The Shapiro–Wilk normality test was employed to evaluate the normality of all data distributions. Between-group comparisons were conducted using Student's t-test for statistical analysis. All statistical tests were performed as two-sided analyses, with statistical significance defined as p < 0.05. Results were graphically presented using the ggplot2 R package for data visualization.

## Results

### Characteristics and analysis of VirHF adverse events

**Fig. 1:**
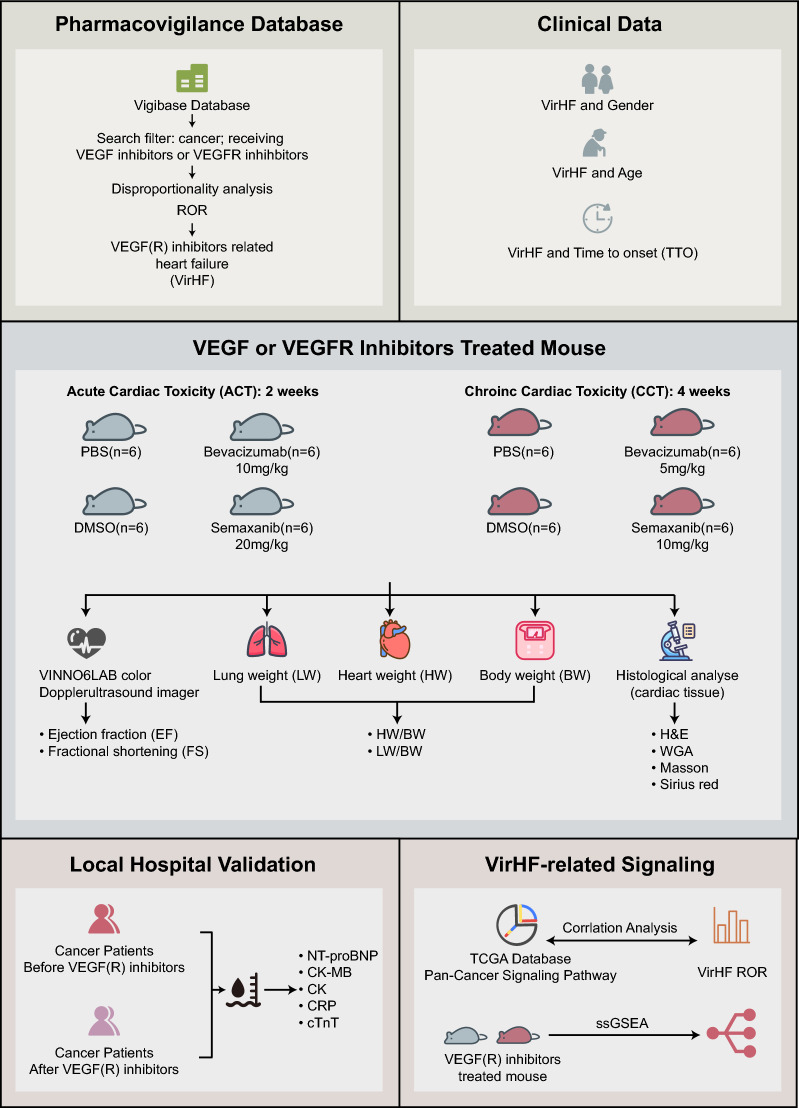
Comprehensive research framework for investigating VirHF. Our investigation began with screening and analysis of clinical characteristics, epidemiological features, and prognostic impacts of VirHF using data from Vigibase, the World Health Organization's global pharmacovigilance database. For clinical validation, we assessed both acute and chronic cardiac toxicity in VEGF(R)i-treated mouse models and investigated underlying molecular mechanisms through comprehensive histopathological analysis and transcriptome sequencing. In a single-center validation cohort, we performed retrospective analyses of cardiac function parameters in patients receiving VEGF(R)i therapy. Using integrated multi-omics analysis and advanced bioinformatics approaches, we elucidated key signaling pathway networks involved in VirHF pathogenesis, thereby advancing our understanding of VEGF(R)i-induced cardiac toxicity. VirHF: VEGF/VEGFR Inhibitor-Related Heart Failure; VEGF(R)i: VEGF or VEGFR inhibitor

**Fig. 2:**
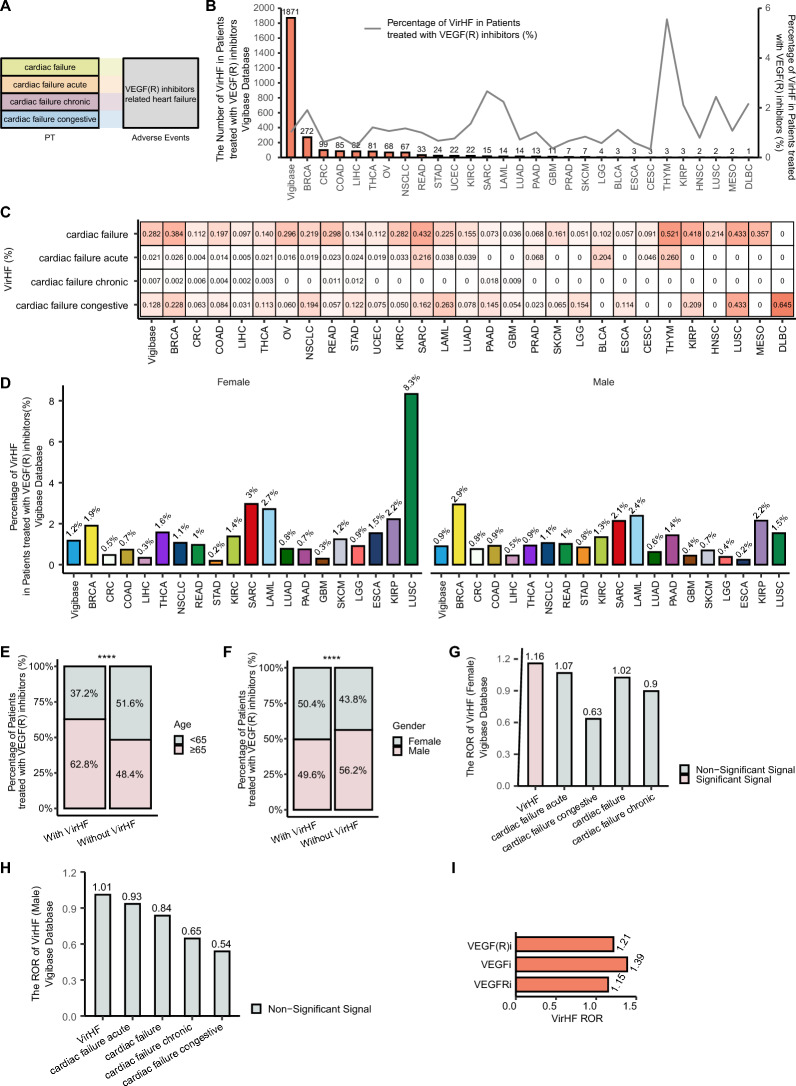
Epidemiological analysis of VirHF characteristics. **A** Sankey diagram depicting the major types of VirHF. **B** Distribution of VirHF cases and their proportions across cancer types. **C** Comparative analysis of VirHF subtype frequencies across cancer types. **D** Gender-specific distribution of VirHF across cancer types. **E** Age-stratified analysis of VirHF incidence: elderly (≥ 65 years) versus younger (< 65 years) patients. **F** Gender-based comparison of VirHF incidence. **G** Sex-specific ROR analysis of VirHF: female cohort. **H** Sex-specific ROR analysis of VirHF: male cohort. **I** Comparative ROR analysis across VEGF(R)i treatment regimens. VirHF: VEGF/VEGFR Inhibitor-Related Heart Failure; ROR: reporting odds ratio; VEGF(R)i: VEGF or VEGFR inhibitor; Significant-Signal: the number of adverse events ≥ 3 and the ROR_025_ > 1; Non-significant Signal: the number of adverse events < 3 or the ROR_025_ ≤ 1; ****p < 0.0001

A systematic analysis of the Vigibase database revealed four distinct types of HF strongly associated with VEGF(R)i. The comprehensive methodology for identifying VirHF cases is illustrated in Fig. 1. Analysis of the Vigibase database identified a total of 1871 VirHF cases (Fig. 2A, B). Of these cases, breast cancer (BRCA) represented the highest proportion with 272 reports, followed by colorectal cancer (CRC; 99 cases), colon adenocarcinoma (COAD; 85 cases), hepatocellular carcinoma (LIHC; 82 cases), and thyroid cancer (THCA; 81 cases). Among the four distinct categories, cardiac failure demonstrated the highest prevalence (0.282%), followed by congestive cardiac failure (0.128%), acute cardiac failure (0.021%), and chronic cardiac failure (0.007%) (Fig. 2C). Sex-stratified analysis demonstrated a higher incidence rate in female patients (1.2%) than in male patients (0.9%). Sex-specific variations in VirHF occurrence rates were observed across different cancer types (Fig. 2D). Subgroup analyses revealed significant differences in VirHF occurrence patterns across various demographic populations. Age-stratified analysis demonstrated that elderly patients (≥ 65 years) exhibited significantly higher susceptibility to HF compared to younger patients (62.8% vs 48.4%; p < 0.05) (Fig. 2E). Sex-based analysis showed significantly higher reporting rates in female patients relative to males (50.4% vs 43.8%; p < 0.05) (Fig. 2F). These findings indicate that age and sex represent significant risk factors for VirHF. Importantly, a significant correlation between VirHF and VEGF(R)i was identified in female patients (Fig. 2G), whereas no such correlation was observed in male patients (Fig. 2H). Analysis of ROR across different treatment regimens confirmed that both VEGFi and VEGFRi are capable of inducing VirHF (Fig. 2I).

### Association of VirHF with gender, age, and clinical outcomes in cancer patients

**Fig. 3:**
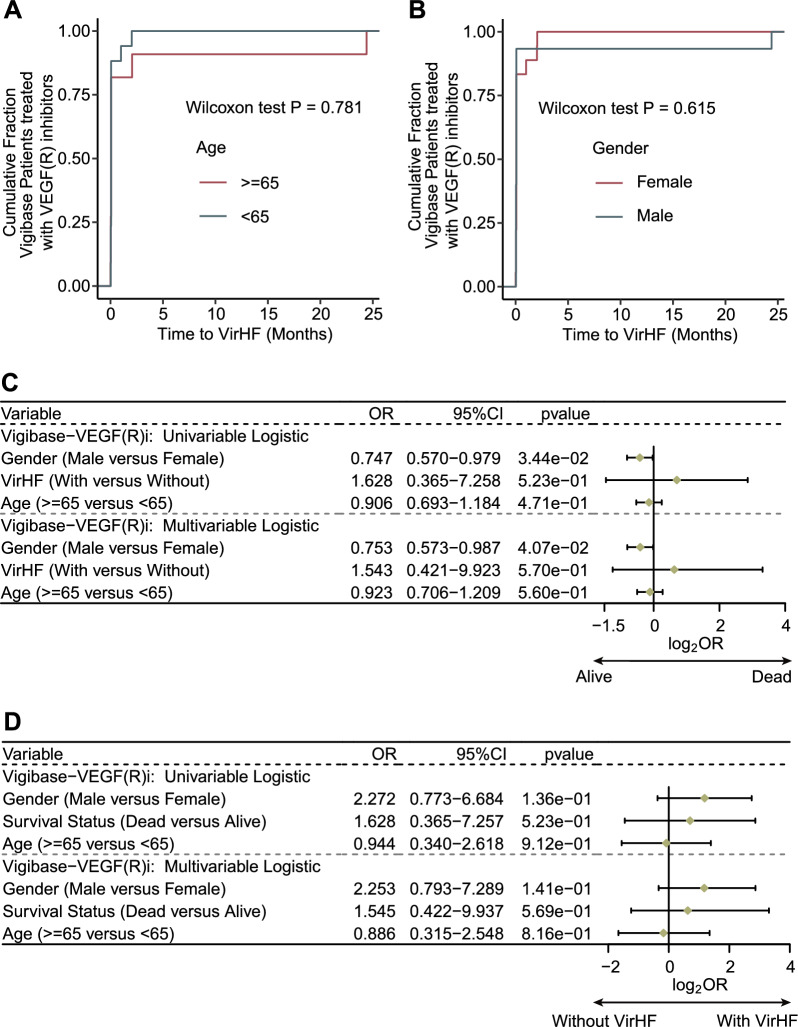
Analysis of the association between VirHF and patient outcomes. **A** Age-stratified TTO analysis of VirHF. **B** Gender-specific TTO analysis of VirHF. **C** Logistic regression analyses (univariate and multivariate) of demographic factors and VirHF impact on patient outcomes. **D** Logistic regression analyses of demographic and survival factors associated with VirHF in cancer patients. VirHF: VEGF/VEGFR Inhibitor-Related Heart Failure; TTO: time-to-onset

Statistical analysis revealed no significant differences in the temporal progression of VirHF across different age groups (p = 0.781, Fig. 3A). Similarly, no statistically significant differences were observed in VirHF onset timing between male and female patients (p = 0.615, Fig. 3B). To systematically evaluate prognostic factors, univariate and multivariate logistic regression analyses were performed to assess the effects of age, gender, and VirHF on patient survival. The regression analyses demonstrated that male gender served as an independent protective prognostic factor (univariate OR = 0.747, multivariate OR = 0.753, both p < 0.05, Fig. 3C). Subsequent analyses demonstrated that age, gender, and survival status were not significantly associated with the risk of VirHF occurrence (p > 0.05, Fig. 3D).

### Molecular mechanisms underlying VirHF

**Fig. 4:**
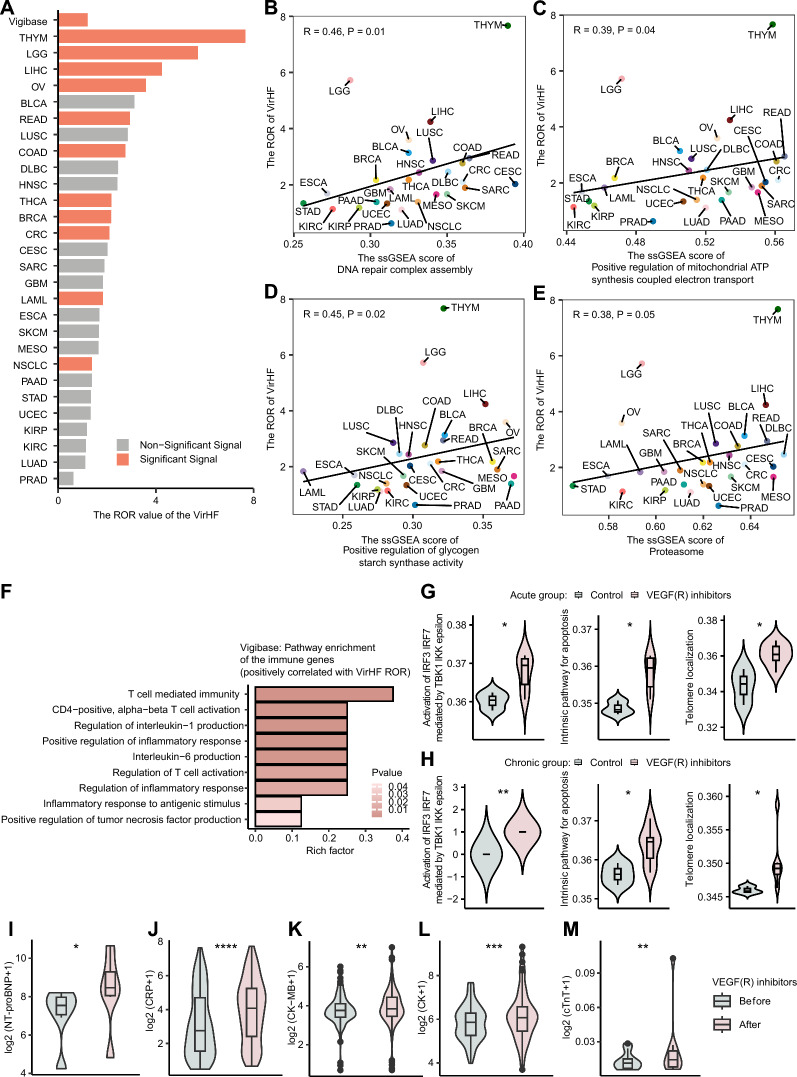
Molecular mechanisms underlying VirHF. **A** Distribution of ROR for VirHF across cancer types: a heatmap analysis. **B** Association between DNA repair pathways and VEGF(R)i-induced cardiac dysfunction. **C** Relationship between mitochondrial ATP synthesis pathways and VEGF(R)i-related cardiac dysfunction. **D** Analysis of glycogen metabolism regulatory pathways in VirHF. **E** Assessment of proteasome pathway activity in VirHF. **F** Gene ontology enrichment analysis of biological processes associated with VirHF. **G** Comparative analysis of signaling pathway activities: acute VEGF(R)i treatment versus control. **H** Comparative analysis of signaling pathway activities: chronic VEGF(R)i treatment versus control. **I** Temporal changes in NT-proBNP levels following VEGF(R)i administration. **J** Temporal changes in CRP levels following VEGF(R)i administration. **K** Temporal changes in CK-MB levels following VEGF(R)i administration. **L** Temporal changes in CK levels following VEGF(R)i administration. **M** Temporal changes in cTnT levels following VEGF(R)i administration. VirHF: VEGF/VEGFR Inhibitor-Related Heart Failure; ROR: reporting odds ratios; VEGF(R)i:; NT-proBNP: N-terminal pro-B-type Natriuretic Peptide; CRP: C-reactive Protein; CK-MB: Creatine Kinase-MB; CK: Creatine Kinase; cTnT: Cardiac Troponin T; Significant-Signal: the number of adverse events ≥ 3 and the ROR025 > 1; Non-significant Signal: the number of adverse events < 3 or the ROR025 ≤ 1

To elucidate the molecular mechanisms of VirHF, we initially evaluated the distribution of ROR for VirHF across diverse cancer types (Fig. 4A), which demonstrated that thymoma (THYM), low-grade glioma (LGG), LIHC, ovarian cancer (OV), and bladder cancer (BLCA) displayed the top five highest ROR values. The ssGSEA performed on the TCGA database revealed significant positive correlations between VirHF ROR values and several key signaling pathways, including DNA repair pathway (R = 0.46) (Fig. 4B), mitochondrial ATP synthesis pathway (R = 0.39) (Fig. 4C), glycogen metabolism regulatory pathway (R = 0.45) (Fig. 4D), and proteasome pathway (R = 0.45) (Fig. 4E) (all p < 0.05).

Gene set enrichment analysis (GSEA) identified several biological processes significantly associated with VirHF (Fig. 4F). These processes encompassed inflammatory responses to antigenic stimuli, inflammatory response regulation, T cell-mediated immunity, T cell activation regulation, CD4 + αβ T cell activation, interleukin-6 and interleukin-1 production pathways, and positive regulation of tumor necrosis factor production. The ssGSEA demonstrated significantly upregulated activity in three key pathways: IRF3/IRF7 activation mediated by TBK1/IKKε (REACTOME), intrinsic apoptotic pathway (REACTOME), and telomere localization (GO) in both acute (Fig. 4G) and chronic (Fig. 4H) VEGF(R)i treatment groups relative to controls.

### Longitudinal assessment of cardiac function parameters before and after VEGF(R)i treatment

We conducted a retrospective analysis of patients receiving VEGF(R)i treatment, with systematic evaluation of dynamic changes in cardiac function parameters before and after therapeutic intervention. Our findings revealed that following VEGF(R)i treatment, serum levels of cardiac biomarkers, including NT-proBNP (Fig. 4I), the inflammatory mediator CRP (Fig. 4J), and myocardial injury markers CK-MB (Fig. 4K), CK (Fig. 4L), and cTnT (Fig. 4M), exhibited significant elevation compared to baseline levels (all p < 0.05).

### Acute cardiac toxicity-induced HF by VEGFi/VEGFRi

**Fig. 5:**
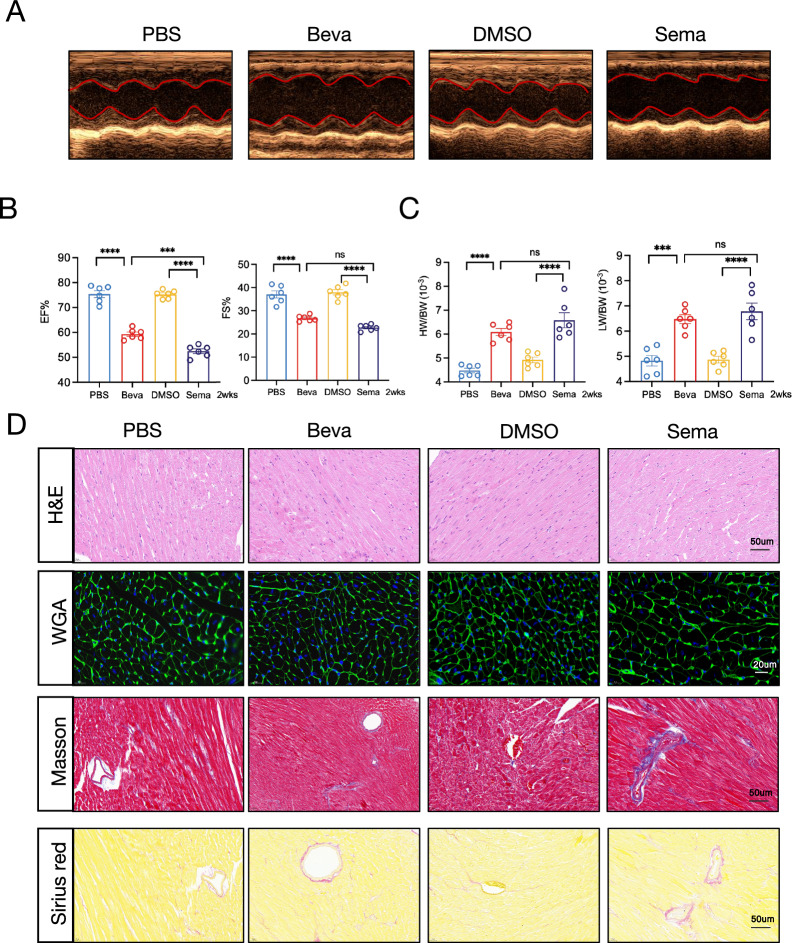
VEGFi/VEGFRi-induced acute cardiac toxicity leading to HF. **A** Representative M-mode echocardiographic images of mice across experimental groups in the ACT model. **B** Quantitative analysis of left ventricular EF and FS across experimental groups in the ACT model. **C** Assessment of cardiac and pulmonary remodeling: HW/BW and LW/BW across experimental groups in the ACT model. **D** Histological analysis of cardiac tissue sections: H&E staining for general morphology (scale bar: 50 μm), WGA staining for cardiomyocyte size (scale bar: 20 μm), Masson’s trichrome staining for fibrosis assessment (scale bar: 50 μm), and Sirius Red staining for collagen deposition (scale bar: 50 μm). Beva, Bevacizumab; Sema, Semaxanib; *p < 0.05; **p < 0.01; ***p < 0.001; ****p < 0.0001; ns: not significant; HF: feart failure; VEGFi: VEGF inhibitors; VEGFRi: VEGFR inhibitors; ACT: acute cardiac toxicity; EF: ejection fraction; FS: fractional shortening; HW/BW: heart weight/body weight ratio; LW/BW: lung weight/body weight ratio; H&E: hematoxylin and eosin; WGA: wheat germ agglutinin

The acute cardiotoxic effects of bevacizumab and semaxanib were assessed following a two-week treatment period. Echocardiographic analysis demonstrated that short-term administration of both bevacizumab and semaxanib resulted in significant cardiac dysfunction compared to controls, with semaxanib inducing a more marked reduction in EF (Fig. 5A, B). Analysis of HW/BW and LW/BW ratios revealed significant elevations in cardiac and pulmonary indices for both VEGF and VEGFRi, with the VEGFR inhibitor exhibiting more substantial increases (Fig. 5C). Histological analyses using H&E, WGA, Masson's trichrome, and Sirius Red staining demonstrated that semaxanib induced more pronounced cardiomyocyte hypertrophy and myocardial fibrosis in the ACT model (Fig. 5D).

### Long-term cardiotoxicity-induced HF by VEGFi/VEGFRi

**Fig. 6:**
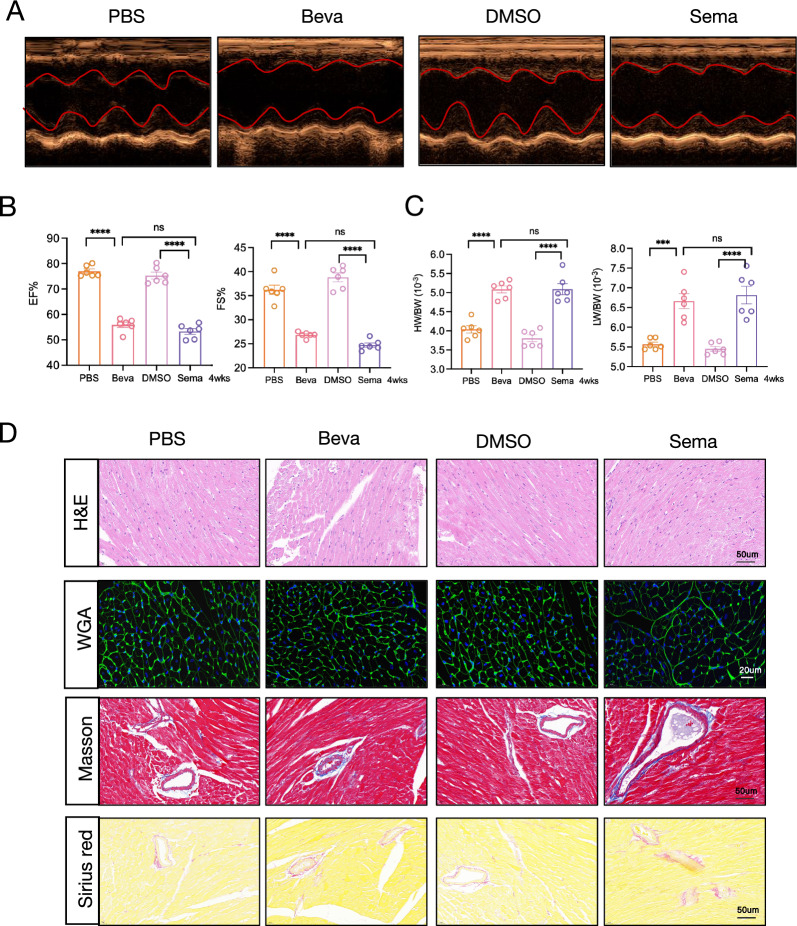
VEGFi/VEGFRi-induced chronic cardiac toxicity leading to HF. **A** Representative M-mode echocardiograms from different experimental groups in the CCT mouse model. **B** Left ventricular EF and FS measurements across experimental groups in the CCT mouse model. **C** HW/BW and LW/BW among experimental groups in the CCT mouse model. **D** Representative cardiac histological analyses of experimental groups in the CCT mouse model: H&E (scale bar: 50 μm), WGA (scale bar: 20 μm), Masson's trichrome (scale bar: 50 μm), and Sirius Red (scale bar: 50 μm). Beva, Bevacizumab; Sema, Semaxanib; *p < 0.05; **p < 0.01; ***p < 0.001; ****p < 0.0001; ns: not significant; HF: heart failure; VEGFi: VEGF inhibitors; VEGFRi: VEGFR inhibitors; CCT: chronic cardiac toxicity; EF: ejection fraction; FS: fractional shortening; HW/BW: heart weight/body weight ratio; LW/BW: lung weight/body weight ratio; H&E: hematoxylin and eosin; WGA: wheat germ agglutinin

The long-term cardiotoxic effects of Bevacizumab and Semaxanib were evaluated over a four-week period. Echocardiographic analysis demonstrated that long-term administration of Bevacizumab and Semaxanib (at half dose) resulted in significant decreases in EF and FS, concurrent with significant increases in cardiac index (HW/BW) and pulmonary index (LW/BW) (Fig. 6A–C). Histopathological analysis of cardiac tissues from CCT model groups revealed persistent pathological cardiomyocyte hypertrophy in mice receiving long-term treatment with Bevacizumab and Semaxanib, although the differential effects on cardiomyocyte hypertrophy between these agents were attenuated. These findings indicate that despite the less severe cardiomyocyte hypertrophy observed in the Bevacizumab ACT model compared to Semaxanib, regular monitoring of cardiac structure and function in patients receiving VEGFi therapy is essential. Moreover, significant cardiomyocyte hypertrophy and fibrosis persisted in the VEGFRi (Semaxanib) CCT group, indicating that VEGFR inhibitor-induced cardiac pathologies, including cardiomyocyte hypertrophy, fibrosis, and cardiac dysfunction, maintain a chronic presence. This underscores the necessity for systematic surveillance of cardiac structure and function in patients undergoing VEGFR inhibitor therapy.

## Discussion

In this study, we conducted the first comprehensive analysis of clinical and epidemiological characteristics of VirHF utilizing VigiBase, the world's largest pharmacovigilance database. Through detailed analysis of VirHF occurrence patterns across multiple cancer types, we identified significant associations between VirHF incidence and demographic factors, particularly patient age and sex. Additionally, we established experimental murine models and implemented transcriptomic analysis to elucidate key molecular pathways involved in VirHF pathogenesis and progression. These findings advance our understanding of VEGF(R)i-induced cardiotoxicity mechanisms. This pioneering global investigation characterizes the clinical profile of VirHF, providing crucial guidance for clinicians in optimizing the benefit-risk assessment of VEGF(R)i therapy.

In our experimental investigations, we systematically characterized the pathophysiological features of VirHF using murine models administered with VEGF(R)i. Our investigations demonstrated that VEGF(R)i administration significantly impaired cardiac function in both acute and chronic treatment protocols, resulting in marked decreases in left ventricular EF and FS. These findings demonstrate strong concordance with VirHF manifestations documented in previous clinical studies [12]. Of particular significance, we established for the first time that VEGFRi (Semaxanib) induced more pronounced cardiac dysfunction than VEGFi (Bevacizumab) in the acute cardiotoxicity model, potentially attributable to VEGFRi's broader kinase inhibition spectrum [2, 9]. Histopathological analysis elucidated the structural mechanisms underlying VirHF. WGA and Masson staining revealed significant cardiomyocyte hypertrophy and myocardial fibrosis in VEGF(R)i-treated groups, which aligned with HF pathological features previously documented in the literature [24]. In the chronic administration model specifically, pathological changes in cardiomyocytes persisted despite dose reduction, indicating that VirHF demonstrates persistent characteristics. This finding carries substantial clinical implications, underscoring the necessity of ongoing cardiac function monitoring during VEGF(R)i therapy.

Molecular analysis revealed multiple signaling pathway networks that contribute to the pathogenesis of VirHF. Notably, aberrant activation of DNA repair-related pathways emerged as a key mediator in the molecular pathogenesis of VirHF. Specifically, DNA single-strand break accumulation in cardiomyocytes initiates the DNA damage response (DDR), which activates the NF-κB signaling pathway and promotes inflammatory factor expression [25]. This mechanistic pathway substantiates the observed abnormal activation of DNA repair pathways identified in the current analysis. Additionally, mitochondrial ATP synthesis pathway dysfunction represents a crucial component in the pathophysiology of VirHF. Cardiac tissue maintains a continuous, high ATP demand, while physiological cardiac ATP reserves can only sustain heartbeat function for several seconds [26]. In the context of pathological HF, cardiac energy metabolism experiences significant remodeling, transitioning from predominant fatty acid β-oxidation to enhanced glucose utilization, resulting in myocardial energy supply–demand imbalance. Additional analyses demonstrated that dysregulation of glycogen metabolism pathways significantly contributes to VirHF development. Mitochondrial pyruvate carrier (MPC) deficiency induces pathological cardiac glycogen accumulation, subsequently precipitating HF [27]. In terms of protein homeostasis regulation, proteasome functional insufficiency (PFI) represents a common characteristic across various cardiac pathological conditions [24]. This observation provides compelling evidence for the abnormal activation of proteasome pathways demonstrated in the present investigation.

The present investigation systematically elucidated the crucial roles of multiple immune-inflammatory signaling pathways in the pathogenesis and progression of VirHF. Accumulating evidence has established that inflammatory factors are significantly elevated in the systemic circulation of chronic HF patients, functioning as key biomarkers for predicting disease prognosis [28]. Elevated expression levels of pro-inflammatory factors, specifically the TNF superfamily, IL-1 family, and IL-6, not only function as important prognostic indicators but also directly mediate the pathological process of myocardial remodeling [29]. In the context of cellular immunity, research has demonstrated that T cells, while sparsely distributed in normal cardiac tissue, extensively infiltrate cardiac tissue during HF [30]. This observation provides critical insights into the immunopathological mechanisms underlying VirHF. Recent studies have identified IRF3/IRF7 as key transcriptional regulators in cardiovascular stress response, exerting significant effects on VirHF progression [31]. At the cellular level, persistent cardiomyocyte apoptosis induces progressive cardiac cell loss, culminating in lethal HF [32]. Telomere dysfunction demonstrates a significant positive correlation with HF risk, consistent with recent findings establishing the association between leukocyte telomere length and cardiac function [33].

This study has several limitations that merit thorough consideration. Primarily, given our substantial reliance on the VigiBase (a spontaneous reporting database), we must acknowledge the inherent constraints of pharmacovigilance data analysis. Data completeness and accuracy potentially exhibit reporting bias, with increased reporting frequencies observed for novel therapeutic agents and severe clinical outcomes. The database demonstrates limitations in comprehensive clinical documentation, particularly regarding therapeutic protocols, concomitant medications, and pre-existing comorbidities—factors that potentially influence HF onset and progression. Although our murine models yielded significant mechanistic insights, extrapolation of these findings to human pathophysiology necessitates methodical evaluation. While there exists a documented 1:1 relationship of genes and proteins between Mus musculus and Homo sapiens for many protein families [34], interspecies variations in pharmacokinetics, cardiac functionality, and physiological adaptations potentially constrain direct translational applications. Furthermore, our experimental systems demonstrate inherent limitations in replicating the multifaceted comorbidities and chronic cardiovascular sequelae characteristic of oncology patients undergoing VEGF(R)i therapy. The temporal constraints of experimental protocols relative to prolonged clinical therapeutic exposure may underestimate the cumulative cardiovascular impact of VEGF(R)i administration. From a clinical standpoint, although we identified concordant alterations in cardiac biomarkers and inflammatory mediators across experimental models and human subjects, validation of these molecular mechanisms necessitates larger-scale prospective clinical investigations. Clinical implementation of real-time molecular surveillance presents significant challenges, while our comprehension of longitudinal cardiovascular outcomes in VEGF(R)i-treated populations remains insufficient. Additionally, the establishment of pathway-specific cardioprotective interventions based on our molecular findings requires validation through rigorously designed clinical trials. Future investigations addressing these limitations should prioritize large-scale, multicenter prospective cohort studies incorporating comprehensive molecular characterization and detailed clinical phenotyping. The identification and validation of clinically relevant biomarkers derived from elucidated pathways, coupled with integration of real-world evidence and experimental data, will be fundamental for optimizing the clinical management of VirHF. Moreover, exploration of mechanism-based cardioprotective strategies derived from this investigation may yield novel therapeutic approaches for preventing or ameliorating VEGF(R)i-induced cardiac dysfunction in cancer patients. Furthermore, future investigations should explore alternative experimental platforms that align with the 3Rs principle (Replacement, Reduction, and Refinement) in biomedical research. Advanced in vitro cardiac models, particularly engineered heart tissue (EHT), represent promising alternatives for preliminary investigations of VEGF(R)i-induced cardiotoxicity. These biomimetic platforms can recapitulate key aspects of human cardiac physiology, including contractile function, calcium handling, and metabolic responses. The integration of EHT with microfluidic systems and real-time monitoring capabilities could facilitate high-throughput screening of cardiotoxicity mechanisms and potential cardioprotective interventions. Additionally, patient-derived induced pluripotent stem cell (iPSC) cardiac models might enable personalized assessment of VEGF(R)i-associated cardiac risks and therapeutic responses. These advanced in vitro approaches, complemented by carefully designed animal studies and clinical investigations, could enhance our understanding of VirHF pathophysiology while promoting more ethical and efficient research practices.

## Conclusions

This study represents the first comprehensive analysis of clinical characteristics and epidemiological patterns of VirHF utilizing VigiBase, the world's largest pharmacovigilance database. Our analysis demonstrated significant associations between VirHF occurrence and patient demographics (age and gender), with distinct manifestation patterns across various cancer types. Using established VEGF(R)i-treated mouse models, we validated that VEGF(R)i administration induces substantial cardiac dysfunction and pathological myocardial remodeling. Comprehensive transcriptomic analyses identified several critical signaling pathways implicated in VirHF pathogenesis, encompassing DNA repair mechanisms, mitochondrial ATP synthesis, glycogen metabolism regulation, and immune-inflammatory cascades. These observations substantially advance our understanding of VEGF(R)i-induced cardiotoxicity mechanisms while establishing crucial theoretical frameworks for personalized therapeutic approaches and cardiotoxicity prevention strategies in clinical settings. Further investigations are warranted to develop targeted intervention strategies based on these molecular mechanisms, ultimately optimizing the safety profile of VEGF(R)i therapeutics in clinical practice.

## Data Availability

The datasets supporting the conclusions of this article are included within the article and its additional files.
